# Views and opinions of patients with glaucoma and age-related macular degeneration on vision home-monitoring: a UK-based focus group study

**DOI:** 10.1136/bmjopen-2023-080619

**Published:** 2024-07-12

**Authors:** Sonali Dave, Mehal Rathore, Peter Campbell, David F Edgar, David P Crabb, Tamsin Callaghan, Pete R Jones

**Affiliations:** 1 Department of Optometry and Visual Sciences, School of Health and Psychological Sciences, City, University of London, London, UK; 2 Royal Free Clinical Research Facility, Royal Free London NHS Foundation Trust, London, UK

**Keywords:** glaucoma, telemedicine, medical retina

## Abstract

**Abstract:**

**Objective:**

To investigate the views, hopes and concerns of patients living with glaucoma and age-related macular degeneration (AMD) regarding vision home-monitoring.

**Design:**

Qualitative study using focus groups and questionnaires. Participants were given three disease-relevant home-monitoring tests to try. The tests consisted of three visual field tests for the glaucoma groups (Melbourne Rapid Fields, Eyecatcher, Visual Fields Fast) and three acuity and/or contrast-sensitivity tests for AMD groups (Alleye, PopCSF, SpotChecks). Focus group data were thematically analysed.

**Setting:**

University meeting rooms in London, UK.

**Participants:**

Eight people with glaucoma (five women, median age 74) and seven people with AMD (four women, median age 77) volunteered through two UK-based charities. Participants were excluded if they did not self-report a diagnosis of glaucoma or AMD or if they lived further than a 1-hour travel distance from the university (to ensure minimal travel burden on participants).

**Results:**

Six themes emerged from focus groups, the two most frequently referenced being: ‘concerns about home-monitoring’ and ‘patient and practitioner access to results’. Overall, participants believed home-monitoring could provide patients with a greater sense of control, but also expressed concerns, including: the possibility of home-monitoring replacing face-to-face appointments; the burden placed on clinicians by the need to process additional data; struggles to keep up with requisite technologies; and potential anxiety from seeing worrying results. Most devices were scored highly for usability, though several practical improvements were suggested.

**Conclusion:**

Patients with mild-to-moderate glaucoma/AMD expect vision home-monitoring to be beneficial, but have significant concerns about its potential implementation.

**Strengths and limitations of this study**

- A strength of this study design was that participants were able to try some of the home monitoring devices. This allowed participants to be informed about what home monitoring devices might look like, and to provide their opinions based on their experiences.
- Another strength of this study is the fact that patients with glaucoma and age-related macular degeneration (AMD) were included. This increases the generalisability of the study to two patient populations.
- However, the generalisability of the study is still limited by the fact that the participants were all volunteers with access to technology (as they were contacted by email). These patients may not represent a ‘typical’ patient with glaucoma or AMD.
- The study also took place in a university setting under supervision from the researchers, which does not represent the real-life aspect of home monitoring, where patients would be expected to navigate the tests relatively independently.

## Introduction

 By 2040, the number of people worldwide living with glaucoma or age-related macular degeneration (AMD) is expected to rise from 268 to 400 million.^1 2^ Both conditions require regular monitoring (eg, yearly,^3^ or twice yearly,^4 5^ or sometimes even more frequently^6^). This monitoring will place increasing strain on hospital eye services, many of which were struggling to meet minimum standards even pre-COVID-19.^7^,^9^

Home-monitoring could help alleviate the strain. Its proponents claim variously that it could provide more efficient, intelligent patient management, produce more reliable, timely patient data and/or free-up appointment availability for high-priority cases.^10 11^

However, evidence for the practical viability of ophthalmic telemedicine remains lacking. Most studies validating home-monitoring devices collect data under artificial, ‘laboratory’ conditions.^12^,^14^ And those few studies that have asked patients to make longitudinal measurements at home^15^,^18^ have generally focused on the accuracy and reliability of the core technologies, without considering wider structural questions including affordability, feasibility or patient acceptability.

Careful reading of the literature does, however, hint at potential concerns regarding the real-world viability of home-monitoring. For example, while Hu *et al* (2023) showed that many patients with glaucoma consider home perimetry and tonometry to be both acceptable and useful, in practice 25% of patients needed to contact the researchers for assistance during the study, and 1 in 20 participants were excluded altogether due to ‘an inability to demonstrate competence’. Similarly, Guigou *et al* (2021) investigated AMD home acuity assessments, and found that by the end of the 9-month study period only 24% of participants remained active users.^16^

The present study used focus groups to qualitatively explore patients’ views regarding the acceptability of home-monitoring, and its perceived challenges and opportunities. Participants were shown a range of potential, disease-appropriate home-monitoring devices, to avoid focusing on any one specific technology. And we included patients with both glaucoma or AMD, to try to generalise beyond a single patient population.

## Methods

### Participants

15 people with established diagnoses of glaucoma (N=8) or AMD (N=7) participated in focus groups at City, University of London between May and June 2022. (Two focus groups for patients with glaucoma. Two for AMD. Three or four people per group.) The groups were intentionally kept small to encourage everyone to participate. Recruitment was through a database of previous research volunteers, and via newsletters of two UK eye charities (Glaucoma UK; The Macular Society). Inclusion criteria were: (1) a self-reported diagnosis of glaucoma or AMD, and (2) the ability to travel to City, University of London. All participants provided written informed consent. The study had ethical approval from the optometry proportionate review committee at City, University of London (#ETH2122-0368) and was conducted in accordance with the Declaration of Helsinki.

### Home-monitoring tests

**Figure 1:**
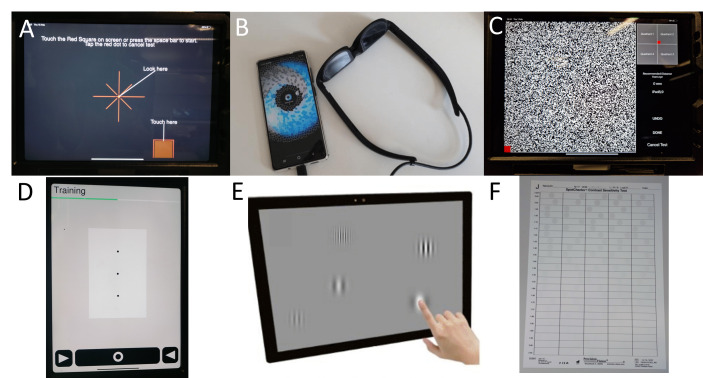
Images showing the tests given to participants in the glaucoma groups (Panels A–C) and the AMD groups (Panels D–F). These were: (**A**) Melbourne Rapid Fields (image from Ref~[^36^]); (**B**) Eyecatcher; (**C**) Visual Fields Fast; (**D**) Alleye; (**E**) PopCSF; and (**F**) SpotChecks.

Participants were given three example home-monitoring technologies to try (though no actual clinical data were collected). Patients with glaucoma were given three visual field tests (figure 1A–C), while patients with AMD were given one hyperacuity test and two contrast sensitivity tests (figure 1D–F). Details of all six tests are given in online supplemental material 1.

### Procedure

The present focus group study used a positivist, qualitative study approach, featuring semi-structured topic guides and facilitated by authors SD and MR. This methodology allowed participants to communicate with each other and explore their views in a comparatively informal setting. Focus groups also encourage participants to engage in open conversation and generate their own questions and analysis from shared experiences.^19^ A copy of the topic guide for the focus groups can be found as supplementary material (online supplemental material 2, table S1Supplementary ).

The predefined topics focused on: participants’ thoughts on the specific tests experienced; thoughts on the concept of telemedicine/home-monitoring in general; how frequently they would be willing to do these tests at home; whether anything would stop them from using the tests, and what would motivate them to keep using the tests.

To contextualise focus group responses, additional steps were taken to roughly characterise: (1) participants’ technical proficiency, and (2) the usability of the home-monitoring technologies. To assess technical proficiency, participants were asked a series of simple questions (‘What technology do you currently own?’, ‘Do you need any help using the technology?’ and ‘From whom do you get help?’). To assess the usability of each device, participants were asked to complete the System Usability Scale (SUS): a general-purpose, Likert-based standardised scale for assessing the perceived usability of digital technologies,^20^ containing statements such as ‘I felt very confident using the system’. All participants completed the SUS once for each device. Finally, to further understand how home-monitoring systems might be improved, participants were presented with six possible features (online supplemental material 3, table S2) and asked to rank them by importance.

### Analysis

Focus group proceedings were transcribed by a professional transcription service (Sterling Transcription Service, London, UK). Thematic analysis was performed using NVivo V.12 (QSR International, Cambridge, Massachusetts, USA), as follows. First, author SD performed framework analysis^21 22^ to identify key themes in each focus group. This consisted of seven steps: transcription, familiarisation, coding, developing an analytical framework, applying the analytical framework, charting data into the framework matrix and interpreting the data. Issues and discussion points from the transcripts were coded into different sections (‘nodes’). The nodes were independently reviewed by another researcher (PRJ) and disagreements were taken to a third researcher (TC) for the final decision. The final agreed nodes were grouped into key themes, which were reviewed by two researchers (PRJ and TC) to create the final framework for analysis. For summaries of all authors bios, please see online supplemental material 4- Author bios.

### Patient and public involvement

After drafting the manuscript, all participants were given a copy of the report and asked to provide feedback on whether the themes and points accurately reflected their views and priorities. Five participants replied, all of whom responded positively, indicating that their views were well represented.

## Results

Eight people with glaucoma and seven with AMD participated (see online supplemental material 5, table S3 for demographics and disease type). All participants had been diagnosed at least 3 years prior to the focus groups (mean 7.4 years) and lived within 1-hour travelling time by public transport/taxi from City, University of London.

**Figure 2:**
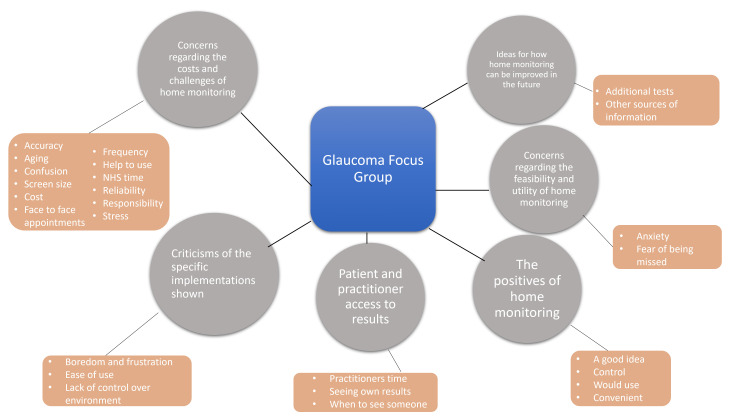
Spider diagram displaying the themes from the glaucoma focus groups. The grey circles represent the themes, and the orange boxes are the subthemes or ‘nodes’. See also online supplemental material 6, figure S1 for a more conventional, bar-chart visualisation of these same data.

**Figure 3:**
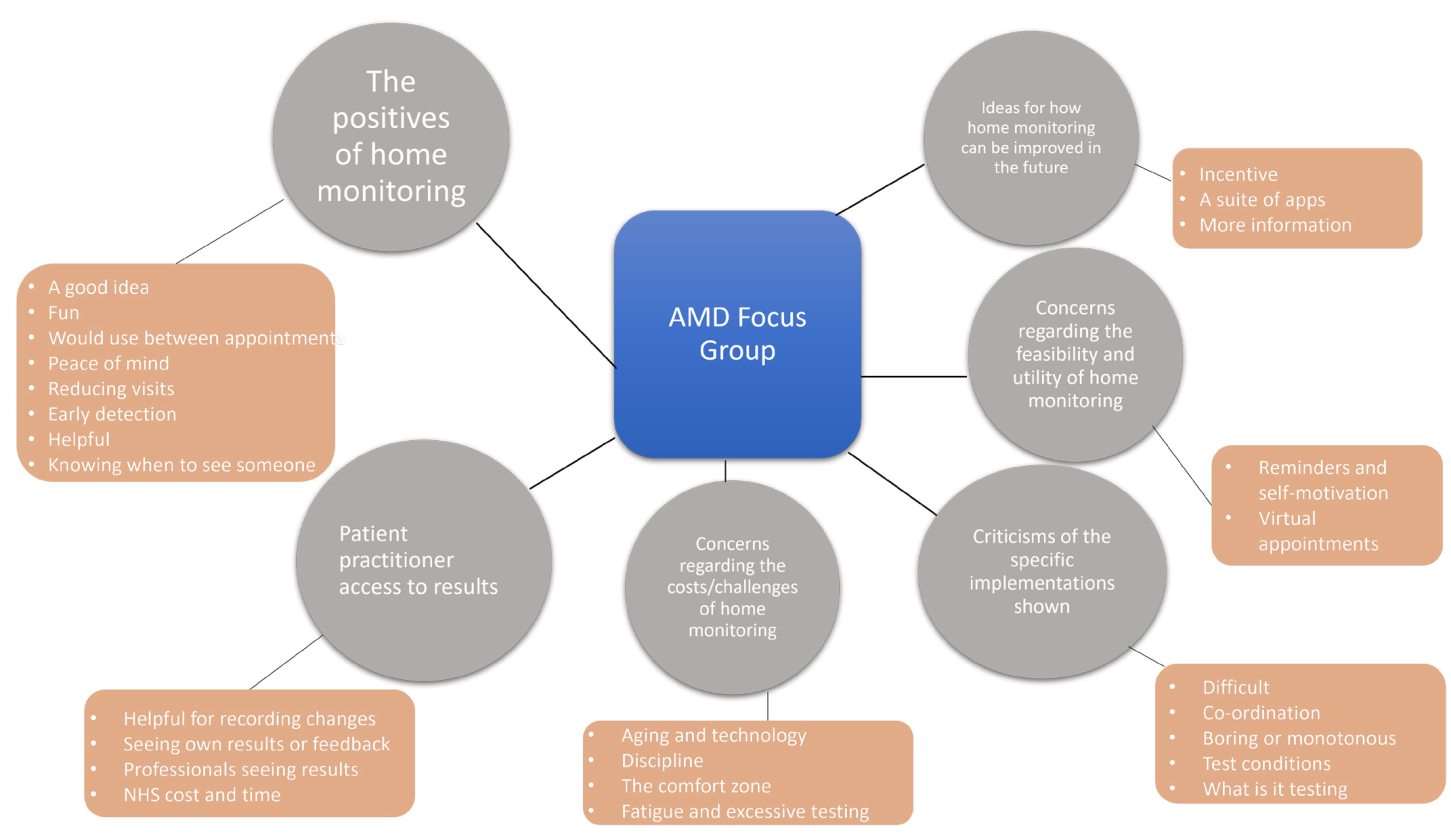
Spider diagram displaying the themes from the AMD focus groups, shown in the same format as figure 2. AMD, age-related macular degeneration.

Six overall themes emerged (figures2  3). The most frequently discussed theme among patients with glaucoma was ‘concerns regarding the costs/challenges of home-monitoring’ (figure 2). The most frequently discussed theme in AMD groups was ‘the positives of home-monitoring’ (figure 3). The following sections present key findings for each theme: Theme 1 was referenced most frequently by all focus groups combined, while Theme 6 was referenced least often.

### Theme 1: concerns regarding the costs/challenges of home-monitoring

The theme with the greatest number of comments overall, was ‘concerns regarding the practical feasibility of home-monitoring’. These comments mostly focused on a number of challenges, delineated below, that participants felt needed addressing before home-monitoring could be implemented effectively.

First, both patient groups expressed concerns that as individuals age, they either may lack access to the requisite technology or may struggle to keep up with the requisite technologies, potentially resulting in changes in patients’ vision being missed:

Of course, it’s relying on, to some extent, the ageing process and then us still being able to manage to do our own testing at home and then we’ve not sort of – for some reason we’re now past it, so to speak. - Glaucoma P3

I think young people, you know, do it, they just cope with everything. Where, for me, I mean I don’t even – I mean, I use my computer simply because I have to, not because I want to. - AMD P1

The other thing is there are quite a fair number of people who probably would find even that little gadget a bit difficult to use, and if the system started depending on people using those devices, some people might really get missed, because they really weren’t quite up to the tech. - Glaucoma P2

I think we must realise in this country that not everybody has a computer, a laptop, or a smart phone, and they certainly don’t know how to use it. But I would say that a lot of very elderly people cannot and don’t want to learn how to use a computer. - AMD P5

To explore further our participants’ access to—and proficiency with—technology, we asked them to record what devices they own. 93% owned a laptop or desktop, 80% a smartphone and 47% of participants owned a tablet. Subsequently, when asked about whether or not they needed assistance with using their technology, 33% said they needed some help, with most helpers being ‘friends and family’.

Second, participants questioned how home-monitoring might impact healthcare costs. All thought home-monitoring would be helpful in reducing the number of hospital visits. But several noted that results would still require scrutiny. Therefore—given the greater potential volume of data—they wondered whether the workload for clinicians might remain unchanged, or even increase:

The only concern is, of course is it being monitored when it gets to the other end, or is it just being sort of filed away for another six months. - Glaucoma P1

But if you’re sending them results every month, they’re going to get overwhelmed. We won’t be saving any money. - AMD P1

Finally, participants were asked how frequently they would be willing to perform these home-monitoring tests, with the concern being testing fatigue. All said they would do the tests as frequently as their clinicians asked them to. However, the glaucoma group did express concerns about fatigue from excessive testing:

I felt tired at my eyes. So doing this every two weeks, for me, might be too often. - Glaucoma P4

On the other hand, AMD participants all said that with reminders, they would be more likely to do the tests when required and therefore the frequency of testing was less of a concern:

I think it’s good because, from my experience, I don’t have any regular check-ups in the diary, so it’s good to force yourself to be aware to check and – because you might not notice. - AMD P1

But if it was a matter of going in to see a clinician, they wouldn’t be able to do it more than once every three months or something, would you? So, you’ve got to – if you do it every two weeks at home that would surely be an advantage, yeah, for those who bother to do it. - AMD P7

In summary, all participants mentioned at least one challenge regarding the feasibility of home-monitoring, the most common being technical impediments and the potential staffing costs for healthcare providers. The question about frequency of testing had mixed responses, with participants agreeing about the fatigue that comes with testing, but also understanding the value and importance of regular monitoring.

### Theme 2: patient and practitioner access to results

In both groups, questions were raised about what would happen to the results once the tests had been completed at home. Two main issues emerged: the level of patient access to their own data, and the integration of telemedicine technologies with clinicians and clinical care.

All participants wanted to be able to see their own results and have an explanation of what their results mean:

Yes [we would like to see our own results], and what it means. - AMD P4

Indeed, some individuals went further and intimated they would be comfortable with using these data themselves to monitor their condition and/or make management decisions:

You see at the moment, one of my eyes I have a visit for every 12 weeks, the other eye is six weeks, and in between I go to another consultant for my eyes. But if I had this and I noticed that the eye which is quite good, and I only go [every] 12 weeks, has suddenly got worse, then I could e-mail the consultant and say, can I come in, or I could go straight to A&E. - AMD P2

I think if you’re better informed then you can make the proper decisions, can’t you? - Glaucoma P1

I think it would be very good, and I like this idea of being able to monitor it. That’s what I think the best beauty is. Not necessarily rushing to see the doctor but be able to monitor it. - AMD P1

However, it was suggested that clear guidance was required regarding how the data were presented, with several individuals favouring a clear ‘thermometer’ or ‘traffic light’ format:

In other words, if you do fall below the number then you must – well, you should come in and be seen. - AMD P7

Furthermore, several participants stressed that they would not want to be solely responsible for evaluating their results, and that it was imperative that their clinician had timely access to the data:

You’d send in the results – you’d see the results yourself and he gets them as well, and you both independently make up your mind whether you need to go and see him, I’d have thought. - AMD P7

I would do it, if like [P6] says, it links up with a clinician who can interpret the results, then I would do it not just randomly on my own. - Glaucoma P5

During discussions, two participants in the glaucoma groups raised the point that waiting for a clinician to evaluate telemedical data could diminish the perceived level of control patients have over their own health:

[In those circumstances home-monitoring] wouldn’t be empowering because you’d still be sat there waiting for somebody else to do something. - Glaucoma P5

Overall, all participants favoured seeing their results, provided there was clear guidance on how to interpret the data, and provided this was not abrogating clinician responsibility, discussed further below (see Theme 5).

### Theme 3: the positives of home-monitoring

All participants believed that, practical challenges notwithstanding, home-monitoring could improve healthcare by augmenting existing, in-person appointments:

In the gaps between appointments…not instead of…It’s not taking over the appointment. - AMD P1

As presented later (see Theme 5) participants were keen to stress that home-monitoring ought not replace in-person clinical appointments altogether. But some participants thought home-monitoring might nevertheless be helpful in reducing the overall number of hospital visits:

Within reason. If you can cut down the visits to once a year rather than once every three months that’s reasonable, isn’t it? - AMD P2

Well you could probably space [out] appointments a bit more, which would mean that the system would work better for everybody. - Glaucoma P2

That’s also less time-consuming for the NHS, then that would be good. - Glaucoma P3

Participants also reported that such benefits might be particularly opportune for individuals in remote geographical locations, for whom attending hospital appointments is costly:

If you were out in say a rural area, where I was brought up, it would be a whole day to go to hospital. I mean now it takes me a good hour or more. So, you need transport, whereas if you were monitoring at home, it would give you peace of mind, wouldn’t it?- AMD P4

Participants also discussed other potential benefits of home-monitoring, such as the tests providing patients with more information to discuss with their clinicians during clinical visits:

The fact that you’ve got something you can do at home and you can do it before you see a consultant, I think that’s a big benefit because I’ve had conversations with a consultant where he’s said, well we didn’t do your visual fields so I’ve got nothing to compare it [with]. - Glaucoma P6

You’re reminded usually when your yearly appointment is, so if they say well, is there anything else you want to look at during this appointment, then you say, I would like to look at the results of my [home-monitoring tests]. - AMD P5

Additionally, both groups made the point that home-monitoring would be beneficial in facilitating early detection of changes in visual function. For example, one participant mentioned the benefit of home-monitoring tests allowing young people to check their eyes frequently:

I think it would be a good thing if people were taught to check their eyes from quite a young age, because since I’ve had problems with my eyes, I’ve got my nieces and nephews to check their eyes…and the quicker it’s picked up, the quicker they can save your eyesight. - AMD P2

Participants in the glaucoma group discussed the importance of home-monitoring in helping patients feel they are in control of their condition:

I think being able to have something that enables you to do your own testing and monitoring is really empowering and I would love to have something like that. - Glaucoma P5

I mean the other thing is it gives you a feeling of being in control as well, doesn’t it? So if you acquire one of those things and you feel there is something wrong and contact them. - Glaucoma P1

The eyes are the most important part of your body – somebody’s body, [laughs] so you don’t [want] to lose your sight. So you want to be able to manage it properly. - Glaucoma P4

Finally, participants in both groups believed home-monitoring was a good idea and they would use it if they were advised to do so by their practitioner.

Yes, if I was advised by my ophthalmologist to do it, then yes I would. - Glaucoma P2

I would love to have something to measure visual fields and eye pressure that I could monitor at home. - Glaucoma P5

Overall, although there were a number of positive comments (eg, regarding increased control), these fewer in number than the negative comments made about home-monitoring.

### Theme 4: criticisms of the specific implementations shown

The following comments were made regarding the three specific tests that each participant experienced (see figure 1). In the glaucoma group, participants questioned the potential (in)accuracy of the tests and expressed a general lack of confidence in the notion of home-assessments:

At the moment I don’t feel confident that I would be getting really accurate results. I don’t think I would really want to use any of these tests unless on a very occasional basis, but I don’t see me pursuing these tests at home [at] the moment. - Glaucoma P1

I felt the testing needed some sort of refinement. I just felt that it wasn’t quite complete in some way. - Glaucoma P2

[I] would be really sceptical about trying anything that isn’t as accurate as things you have already. So anything not as good, I don’t really see the point of using them. - Glaucoma P8

However, as many of the study participants have had their diagnosis for a number of years, they said this response may reflect their familiarity with tests done in the clinic, so deviating from these would cause confusion:

I wasn’t used to it. I’m so conditioned by the Humphrey visual field [test], it’s like anything that detours from that. I don’t like change and I’m just used to doing that. - Glaucoma P4

Similarly, despite limited references to this theme in the AMD group, one criticism was that other issues, such as poor co-ordination, may cause them to have a lower score:

You had to be more dextrous because you were having to get your finger up and down the page…and it depends how quick you are, I think…you could miss quite a few, I think. - AMD P2

Another issue from the AMD group was that the tests varied markedly in difficulty depending on the stage of macular degeneration and, thus, may not be appropriate for patients across the disease spectrum. For example, one participant with unilateral wet AMD said:

I found the middle one with the bubbles [PopCSF] really very difficult because they were so light, and I find with my macular degeneration I need sharp objects to see them. - AMD P4

However, two AMD tests were measuring contrast sensitivity which would explain why some participants had difficulties with the test.

In the glaucoma group, some concerns were raised about a lack of control of the testing environment due, in particular, to the nature of visual field testing being dependent on low light conditions. Participants expressed that it would be better to perform the tests in darkness, similar to clinical environments.

Criticisms of [Eyecatcher] would be that I want an immersive thing that it can just block out everything else that’s going on around me. So almost like I’m on a VR headset so I’ve got nothing else that I can see out past there. - Glaucoma P1

[For all of the tests] being able to block out the rest of the light would be much better. - Glaucoma P2

Finally, all participants expressed frustration and confusion with the Visual Fields Fast test, an unconventional noise-campimeter (see figure 1C):

I mean I was wondering whether I understood it properly while I was using it – actually, what am I looking for here? Where’s the dot – you know what a dot is? - Glaucoma P5

Yeah, so perhaps it was perhaps a bit too difficult to see the anomalies. It was a bit too difficult to figure out what you were looking for exactly. - Glaucoma P5

**Figure 4:**
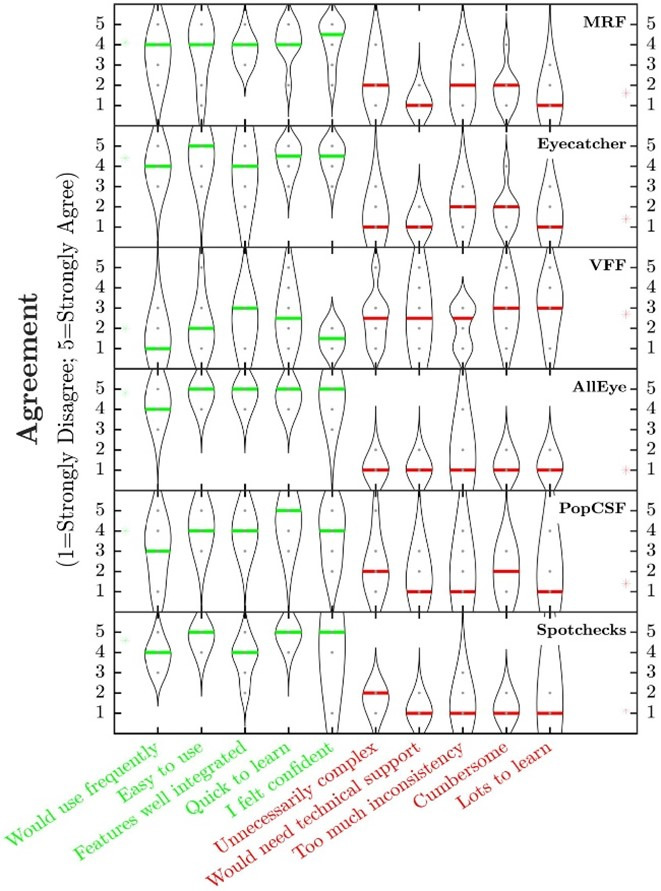
Violin plots showing how each test was rated on the System Usability Scale (MRF: Melbourne Rapid Fields; VFF: Visual Fields Fast). The green font represents positive statements, and the red represents negative statements. The higher a test is rated on the green text statements, and lower on the red statements, the more the test is preferred.

To explore further the usability of each test, participants were asked to rate each using the SUS (see Methods). Overall, ratings were generally high on the five positive statements (see figure 4). The Melbourne Rapid Fields (MRF) and Eyecatcher, on average, scored highly (4.1/5) in the glaucoma group, while in the AMD group all three measures were rated highly on average on the positive statements, with Alleyes scoring highest (4.8/5) followed by SpotChecks (4.6/5) and PopCSF (4/5).

Overall, participants felt that more work was required to refine current tests. The glaucoma group expressed more concerns with frequency of testing and testing environment, whereas the AMD group were more concerned about differing abilities and other issues that may affect performance.

### Theme 5: concerns regarding the feasibility and utility of home-monitoring

Concerns about missing appointments or home-monitoring replacing face-to-face visits were mainly expressed in the AMD group:

I look forward to seeing the consultant and I wouldn’t want to do that online because this is a serious condition. - AMD P3

Anxiety was expressed in the glaucoma group only and included that, without feedback or supervision, they may perform the test incorrectly:

The anxiety of doing it just at home and there’s nobody, it’s like there’s no-one coming to see what I’m doing. - Glaucoma P1

There were also concerns that the results—even if correct—may be disheartening:

I didn’t think I got very good results, that might concern me. - Glaucoma P5

I feel probably if your eye condition was getting worse and worse you might not want to know that. - Glaucoma P8

Finally, glaucoma group members were concerned that home-monitoring might shift the burden of responsibility from clinicians to patients:

I wouldn’t want to be reliant for it to be me only, that I’m responsible for the results and interpreting them and stuff like that. - Glaucoma P7

Participants also discussed fears about home-monitoring which, if poorly implemented, could lead to worsening of existing services, ultimately leading to poorer patient care:

But the only trouble is it can become a sort of way in which the system – I mean the system misses you, because you’re so busy testing yourself at home and thinking things are all right, and the health system may be seeing that’s all right, he’s checking himself at home. But really, if you had actually been visiting the clinic, you might have had something further – further test or investigations done and maybe something could be caught. - Glaucoma P1

I don’t know, it seems to be a two-tier society and I think some people will get left behind by this if telemedicine is treated as the norm. There’s still a need for people to go there and be looked [at]. - Glaucoma P8

But I mean I think the anxiety around it is that the system is trying to fob me off with this piece of equipment, in the hope that I won’t bother them so much. - Glaucoma P4

In summary, the main concern for patients with AMD is that the face-to-face elements of appointments should not be replaced. Participants believed that seeing a clinician face-to-face or even having a phone discussion is very important, providing benefits beyond results screening and should not be completely replaced.

### Theme 6: ideas for how home-monitoring measures could be improved in the future

Participants in all focus groups expressed ideas about what they would like to see from home-monitoring devices in the future. In the glaucoma group, participants felt it was important to have a system alerting them to contact someone:

Well, I think an in-built warning system would be beneficial, apart from your own viewing the documents or results. - Glaucoma P2

If they had a phone line where you could ring and you spoke to somebody, a sort of triage system where they could go, yeah, okay, these are your results. Don’t worry that’s not a concern, keep monitoring or they could say, no come in tomorrow or now. - Glaucoma P6

Similarly, participants in the AMD group all liked the traffic light system in one test which helped to interpret results and gave an alert when they should see a practitioner:

The one where you had to line up the dots, it gives you a score at the end, and the score will either appear in green if you’re doing better than your previous one, it will appear in yellow if you’ve kept it the same, or it will appear in red if you’ve done worse than your previous one. - AMD P2

Participants in the glaucoma focus group asked whether home-monitoring of visual function could be complemented by measurement of intraocular pressure:

Would we ever be able to do eye pressure testing at home? Is that something that’s on the cards? - Glaucoma P4

All participants in the AMD group expressed the importance of having reminders to complete the tests, drawing from their experiences of not using the Amsler grids as regularly as they should:

I think a reminder’s good because, yeah, you’ve got to be self-motivated for anything like that, like exercise or anything that you know you’ve got to do because it’s good for you. - AMD P2

Some participants in the AMD group brought up the idea of having a ‘suite’ of tests, with all the home-monitoring tests recommended by the clinic in one place:

It was only the second one I favoured less, but I still think as a suite, it would be good to have all of them. - AMD P1

Another suggested improvement from the AMD group was to incorporate an incentive for patients to do their testing, such as a spinning wheel that can reward patients for completing their tests:

I’ve not got one of those automatic meters yet, and every month they say they want the record sent in, and if you do that, they give you a free spin on their little magic wheel, and if you win, you’re supposed to get £2 or £5 or something, how about that as an idea? You know, give people some incentive, even though it’s a little bit just to do this thing. - AMD P7

Both groups agreed on the importance of having more information available to them, especially about their own results, other home-monitoring tests and advice:

But I need information, and I need regular testing to know that it’s not getting worse. - AMD P7

There’s so much information out there, it is confusing. But there are respectable sources, I mean, you need to find out about them. - Glaucoma P1

Based on participant comments about what they would like to see in the future, both groups prioritised very different features, but agreed that more information and feedback were of fundamental importance. Participants completed a questionnaire that asked them to rank the importance of features they wished to see in future home-monitoring technologies. The most important feature across both groups was ‘being notified of when to see someone’.

## Discussion

Even within a small cohort of patients with glaucoma and AMD, views regarding vision home-monitoring were mixed. The main positives were that home-monitoring would give patients a greater sense of control of their own condition, and could provide clinical benefits by enabling early detection of disease progression. However, the single most commonly discussed themes were concerns about the costs and challenges of home-monitoring, and concerns regarding access to data (including the cost of requiring clinicians to process large volumes of additional information).

Specific concerns were also raised regarding the tests themselves. Patients generally rated the usability of the technologies as high, as indicated by favourable scores on the SUS. However, they also identified possible design and implementation flaws that could limit adherence or accuracy. They also provided suggestions regarding what they would like to see from home-monitoring devices in the future.

### Reducing the burden on patients and practitioners

Using home-monitoring to augment existing monitoring was universally well received (participants recognised potential logistical and financial benefits). However, participants had mixed views regarding home-monitoring replacing in-person assessments, with concerns raised about possible reduced quality of care (eg, concerns that after being issued a home-monitoring device, patients might be forgotten). They also questioned whether home-monitoring would even reduce the burden on clinicians, noting that the additional data collection might decrease technicians’ current workload, but actually increase clinicians’ workload. Though they also noted that the extra information provided might help clinicians make faster or more accurate decisions^23 24^—something that has also been noted by wider research regarding home-monitoring of other systemic health conditions.^25^

### Patient-practitioner access to results

All participants expressed that, in addition to sending the practitioner their results, they would also want access to their own data. This is consistent with research into other conditions (eg, vascular hypertension), where patients often note that seeing their own results gives them a sense of control^26^ and affords better management of their own health.^27 28^ Though in this respect it is important to note that ophthalmic data is often relatively complex, and it may be significant that all of the participants in the present study had lived with their diagnosis for many years, and so may feel relatively confident interpreting eye test data. Research confirms that patients may struggle with comprehension of complicated medical results,^29 30^ and inaccurate interpretation of results can increase the numbers of hospital visits and levels of anxiety.^31^ Our participants also shared these concerns, particularly that a ‘worse’ result than normal would worry them even though they had been undergoing the tests for a number of years. Interpretation of results could be a much greater concern for ‘newer’ patients.

### Access to technology

One practical obstacle discussed in all focus groups was digital exclusion: the idea that a shift towards home-monitoring might result in some patients ‘missing out’ because they were unable to keep up with the requisite technology. Consistent with this, prior research has found that many patients are put off by digital technologies,^32^ and digital exclusion to have a significant impact on patient care in mental health services, particularly in community services.^33 34^ In this respect, it is interesting to note that even among our sample of reasonably ‘tech savvy’ participants, while a majority owned a computer or smartphone, almost one-third expressed a regular need for assistance to use their devices (typically from friends or family members).

### Comparing the views of patients with glaucoma versus AMD

Participants with glaucoma discussed the positives of home-monitoring less often than those in the AMD groups. The reasons for that are not entirely clear, though one obvious factor is that visual field testing for glaucoma is a significantly longer and more onerous procedure. Additionally, AMD participants are already familiar with the concept of home-testing via the Amsler grid,^35^ and noted that digital technologies could help by providing automated reminders. Conversely, needing reminders was never discussed in the glaucoma group, where the main discussion point was fatigue caused by excessive home testing. Though ultimately most participants agreed they would carry out home-monitoring as often as their physician deemed was required.

### Limitations and future work

The primary limitations of this study relate to generalisability of the sample population. First, participants all lived in highly urban areas. Second, they were highly motivated and self-selecting (being research volunteers). Third, they were contacted via email, necessitating that they possess baseline familiarity with digital technology (though many nevertheless needed regular assistance, as previously noted). Fourth, while their visual impairment was not formally assessed, all participants self-reported their condition as mild-to-moderate and could travel independently, and all had lived with their conditions for some time.

Some of these factors (eg, digital familiarity, high motivation) may have promoted positive opinions about the feasibility of remote monitoring, while others (urban-centric, well-established diagnosis) may have led to particular benefits and opportunities being under-reported. Ultimately, a much larger study is required to understand the acceptability of vision home-monitoring within the patient population more generally, or within specific subgroups (eg, the very elderly). In that sense, the present work should be understood only as an initial exploration of a much wider topic. Though we hypothesise that it is precisely the present patient types (relatively young, self-motivated and with mild-to-moderate disease) for whom home-monitoring is likely to prove most feasible and of greatest utility, short-term.

Regarding the study design, focus group discussions were facilitated using prompts to encourage discussion about the idea of home-monitoring (eg, What do you think about the idea of telemedicine?). Although an established qualitative technique, this is also an inevitable source of potential bias. For example, some prompts used wording that could have led participants to focus on negatives (eg, This application is recommended to be used once every two weeks, would you be likely to do this?) while others could have a focus on positives (eg, Did you think the system was user friendly?). While strenuous efforts were made to maintain impartiality, a key corollary is that readers should not, for example, place too much emphasis on the exact ratio of positive/negative comments.

A final limitation was the setting and limited time that participants had with the range of devices, (around 30 min, under supervision, within a university setting). Despite minimal intervention by the researchers, this does not reflect ‘real-life’ home-monitoring. To achieve this it would be necessary to allow participants to take the devices home for an extended period (eg, as per^15^). Similarly, a future, larger study could examine how patients’ perceptions of home-monitoring vary with disease severity and/or years since diagnosis, or other important practical issues, including the health economics associated with home-monitoring (ie, costs of the test, reimbursement of charges).

## Conclusion

In conclusion, participants with glaucoma and AMD have a number of concerns regarding home monitoring. Despite references to an increased sense of control and the potential for early detection of changes, many participants still felt there was more work to be done. The tests in this study were criticised for some aspects of their design and reliability but participants were clear about what future tests should include. Further work on larger samples can provide more insights based on diagnosis and location.

## supplementary material

10.1136/bmjopen-2023-080619 online supplemental file 1

10.1136/bmjopen-2023-080619 online supplemental file 2

10.1136/bmjopen-2023-080619 online supplemental file 3

10.1136/bmjopen-2023-080619 online supplemental file 4

10.1136/bmjopen-2023-080619 online supplemental file 5

10.1136/bmjopen-2023-080619 online supplemental file 6

## Data Availability

Data are available upon reasonable request.
