# Supplementary material for: Views and opinions of patients with glaucoma and age-related macular degeneration on vision home-monitoring: a UK-based focus group study
Source: BMJ Open. 2024 Jul 12;14(7):e080619. doi: 10.1136/bmjopen-2023-080619 (PMC11253750; doi:10.1136/bmjopen-2023-080619)
Supplement: online supplemental file 1 [file bmjopen-14-7-s001.pdf]

Supplementary materials- IF THIS MANUSCRIPT IS ACCEPTED, ALL SUPPLEMENTAL MATERIAL WILL BE REFORMATTED AS A STANDALONE PDF OR WORD DOCUMENT

Participants were given 3 tests to try, for a total of approximately 30 minutes. To avoid fatigue, participants were instructed that they did not necessarily have to finish each test on both eyes. In practice, for the tests of shorter duration, all participants completed a full test on at least one eye. For the longer tests, some participants completed the test on one eye but others chose to stop the test earlier when they had established a clear idea of what the test involved.

Home-monitoring tests for glaucoma

The participants in the two glaucoma focus groups were given three different Visual Field tests to experience, as shown in **Figure 1** of the main manuscript. The three tests were as follows:

Melbourne Rapid Fields (https://www.appviewmrf.com/), shown in **Figure 1A**, is an iPad-based static suprathreshold perimeter (42). Participants were asked to focus on a fixation point (that periodically shifts location) and to tap a red square at the bottom of the screen when they saw the stimulus, which is presented at varying luminance levels, appear on the screen. Tests are performed monocularly in a dimly lit room and a full test for a single eye lasts approximately four minutes.

Eyecatcher v3 (https://www.eyecatchervision.com), shown in **Figure 1B**, is a portable static threshold perimeter that uses ‘smartglasses’ connected to a smartphone. The test is a new version of a tablet-based perimeter described previously (43, 44): updated to take into account previous feedback from patients (15). As in Standard Automated Perimetry, participants were asked to fixate on a central cross, and press a button whenever they saw a stimulus (200 msec Goldmann III dots of varying luminance). Tests are performed monocularly in a dimly lit room (both eyes interleaved). A full test generally lasts approximately seven minutes (for both eyes).

Visual Fields fast (https://visualfieldfast.com/), shown in **figure 1C**, is a tablet-based white-noise campimeter. Participants were asked to focus on a fixation point (that periodically shifts location) and to draw with their finger any areas of the screen that do not flicker or

which otherwise look abnormal (e.g., scintillation). Tests are performed monocularly in a dim room and a full test for a single eye generally lasts approximately five minutes.

Home-monitoring tests for AMD

The participants in the two AMD focus groups were given one hyperacuity test and two contrast sensitivity tests to experience, as shown in **Figure 1D-F** of the main manuscript. The three tests were as follows:

AllEye (https://alleye.io/), shown in **Figure 1D**, is a tablet-or-smartphone test of near-vision hyperacuity (45). Participants were asked to align the three dots on their screen by pressing arrows on the screen. Participants were presented with three dots at different angles and alignments a total of 12 times. Tests are performed monocularly in a dimly lit room. For a full test, a single eye test generally lasts five minutes.

PopCSF, shown in **Figure 1E**, is an iPad-based task measure of the contrast sensitivity function (i.e., contrast sensitivity and acuity) (46). Participants were asked to 'pop' bubbles (i.e. Gabor patches of varying contrast and spatial frequency) by pressing them as they bounce around the screen. Infrared head-tracking (using the iPad's TrueDepth camera) is used to scale the stimuli based on viewing distance. Tests are performed monocularly in a dim room, with a full test for a single eye lasting approximately five minutes.

Spotchecks (https://www.precision-vision.com/products/contrast-sensitivity-tests/peak-contrast-sensitivity/spotchecks/spotchecks/), shown in **Figure 1F**, is a single-use pen-and-paper contrast sensitivity test (the outcome of which is similar to that of the Pelli Robson letter chart (47)) (48). Participants were asked to mark with an 'x' any dark spots (the contrast of which progressively decreases down the page). Tests are performed monocularly in a dim room, and a full test for a single eye lasts between one and ten minutes, depending on the participant.
