# Supplementary material for: Views and opinions of patients with glaucoma and age-related macular degeneration on vision home-monitoring: a UK-based focus group study
Source: BMJ Open. 2024 Jul 12;14(7):e080619. doi: 10.1136/bmjopen-2023-080619 (PMC11253750; doi:10.1136/bmjopen-2023-080619)
Supplement: online supplemental file 2 [file bmjopen-14-7-s002.pdf]

Supplementary materials- IF THIS MANUSCRIPT IS ACCEPTED, ALL SUPPLEMENTAL MATERIAL WILL BE REFORMATTED AS A STANDALONE PDF OR WORD DOCUMENT

1	What are your overall thoughts on the system? (Prompts: did you think the system was user friendly? Would you recommend this to a friend? Would you use it?)
2	What do you think about the idea of telemedicine ? (Prompts: Do you like the idea of being able to do the tests at home? What do you think are the advantages/disadvantages of telemedicine?)
3	This app is recommended to be used once every two weeks, would you be likely to do this?
4	What would make you stop using them? What would discourage you from using them?
5	How often would you use these apps/systems? (Prompts: Every week? Twice a week? Never? What would motivate you to maintain your use of them?)

Table S1- Focus group conversation topic guide used by the focus group facilitators
