# Supplementary material for: Views and opinions of patients with glaucoma and age-related macular degeneration on vision home-monitoring: a UK-based focus group study
Source: BMJ Open. 2024 Jul 12;14(7):e080619. doi: 10.1136/bmjopen-2023-080619 (PMC11253750; doi:10.1136/bmjopen-2023-080619)
Supplement: online supplemental file 3 [file bmjopen-14-7-s003.pdf]

Supplementary materials- **IF THIS MANUSCRIPT IS ACCEPTED, ALL SUPPLEMENTAL MATERIAL WILL BE REFORMATTED AS A STANDALONE PDF OR WORD DOCUMENT**

**Features**
I would like the app/device to send me text reminders to do the test
I would like the app to read the instructions out loud
I would like to see my results at the end of the test
I would like the app to tell me if there has been a change in my score and if I need to see my practitioner
I would like to receive confirmation that my results have been seen by my practitioner
I would like to be supervised doing the test by my practitioner

Table S2- Table listing the statements presented to participants during the focus group session. Participants were asked to rate these statements from most to least important.
