# Supplementary material for: Views and opinions of patients with glaucoma and age-related macular degeneration on vision home-monitoring: a UK-based focus group study
Source: BMJ Open. 2024 Jul 12;14(7):e080619. doi: 10.1136/bmjopen-2023-080619 (PMC11253750; doi:10.1136/bmjopen-2023-080619)
Supplement: online supplemental file 4 [file bmjopen-14-7-s004.pdf]

Supplementary materials- IF THIS MANUSCRIPT IS ACCEPTED, ALL SUPPLEMENTAL MATERIAL WILL BE REFORMATTED AS A STANDALONE PDF OR WORD DOCUMENT

- Author SD is a fourth year PhD student in the Department of Optometry and Visual Sciences. She has experience with conducting qualitative research and facilitating focus groups as well as the analysis of the data.
- Author MR is a doctoral student and early-career researcher at City, University of London. Her current research work primarily focuses on alternative approaches to visual function testing for age-related eye conditions. She is an optometrist by qualification and her research interests include ocular diagnostics, community eye care and glaucoma.
- Author PC is a senior lecturer in Optometry and Vision Sciences and a practising optometrist. His current areas of research include home monitoring and the use of technology in glaucoma medication adherence.
- Author DE was a practicing optometrist for 35 years and is currently Emeritus Professor at City, University of London. The detection and management of glaucoma has been a major research interest and regular teaching topic for DE for many years. DE's research experience includes both quantitative and qualitative research.
- Author DC is a professor of statistics and vision research. He is an internationally recognised expert in glaucoma management. The research in his lab (Crabb Lab) is a hub of innovation and interdisciplinary health research, focused on improving ocular healthcare for the future.
- Author TC is the Involvement and Inclusion Manager for Research at The Royal Free London hospital. A qualified Optometrist with over 20 years of clinical experience she was previously a Lecturer at City, University of London for 8 years, where she holds an honorary research position. TC specialises in clinical research and has completed similar qualitative and focus group studies previously.
- Author PJ is a lecturer in Optometry and a vision scientist interested in the development of new digital sight tests. His research focuses on developing digital vision tests for heard to reach populations that are pragmatic and easy to use.
