# Supplementary material for: Views and opinions of patients with glaucoma and age-related macular degeneration on vision home-monitoring: a UK-based focus group study
Source: BMJ Open. 2024 Jul 12;14(7):e080619. doi: 10.1136/bmjopen-2023-080619 (PMC11253750; doi:10.1136/bmjopen-2023-080619)
Supplement: online supplemental file 5 [file bmjopen-14-7-s005.pdf]

Supplementary materials- IF THIS MANUSCRIPT IS ACCEPTED, ALL SUPPLEMENTAL MATERIAL WILL BE REFORMATTED AS A STANDALONE PDF OR WORD DOCUMENT

Participant number	Age	Diagnosis
**AMD P1**	80	Unilateral wet AMD
**AMD P2**	73	Bilateral dry AMD
**AMD P3**	84	Bilateral wet AMD
**AMD P4**	93	Unilateral wet AMD
**AMD P5**	77	Bilateral dry AMD
**AMD P6**	49	Bilateral dry AMD
**AMD P7**	77	Bilateral dry AMD
**Glaucoma P1**	79	Primary open angle glaucoma
**Glaucoma P2**	85	Normal tension glaucoma
**Glaucoma P3**		Primary angle closure glaucoma
**Glaucoma P4**	77	Normal tension glaucoma
**Glaucoma P5**	65	Primary angle closure glaucoma
**Glaucoma P6**	70	Normal tension glaucoma
**Glaucoma P7**	68	Primary open angle glaucoma
**Glaucoma P8**	50	Primary angle closure glaucoma

Table S3: Table showing participant demographic and diagnosis information. Glaucoma participant 3 was unable to be contacted for their age and therefore this information could not be presented.
