# Supplementary figures and images for: Views and opinions of patients with glaucoma and age-related macular degeneration on vision home-monitoring: a UK-based focus group study
Source: BMJ Open. 2024 Jul 12;14(7):e080619. doi: 10.1136/bmjopen-2023-080619 (PMC11253750; doi:10.1136/bmjopen-2023-080619)

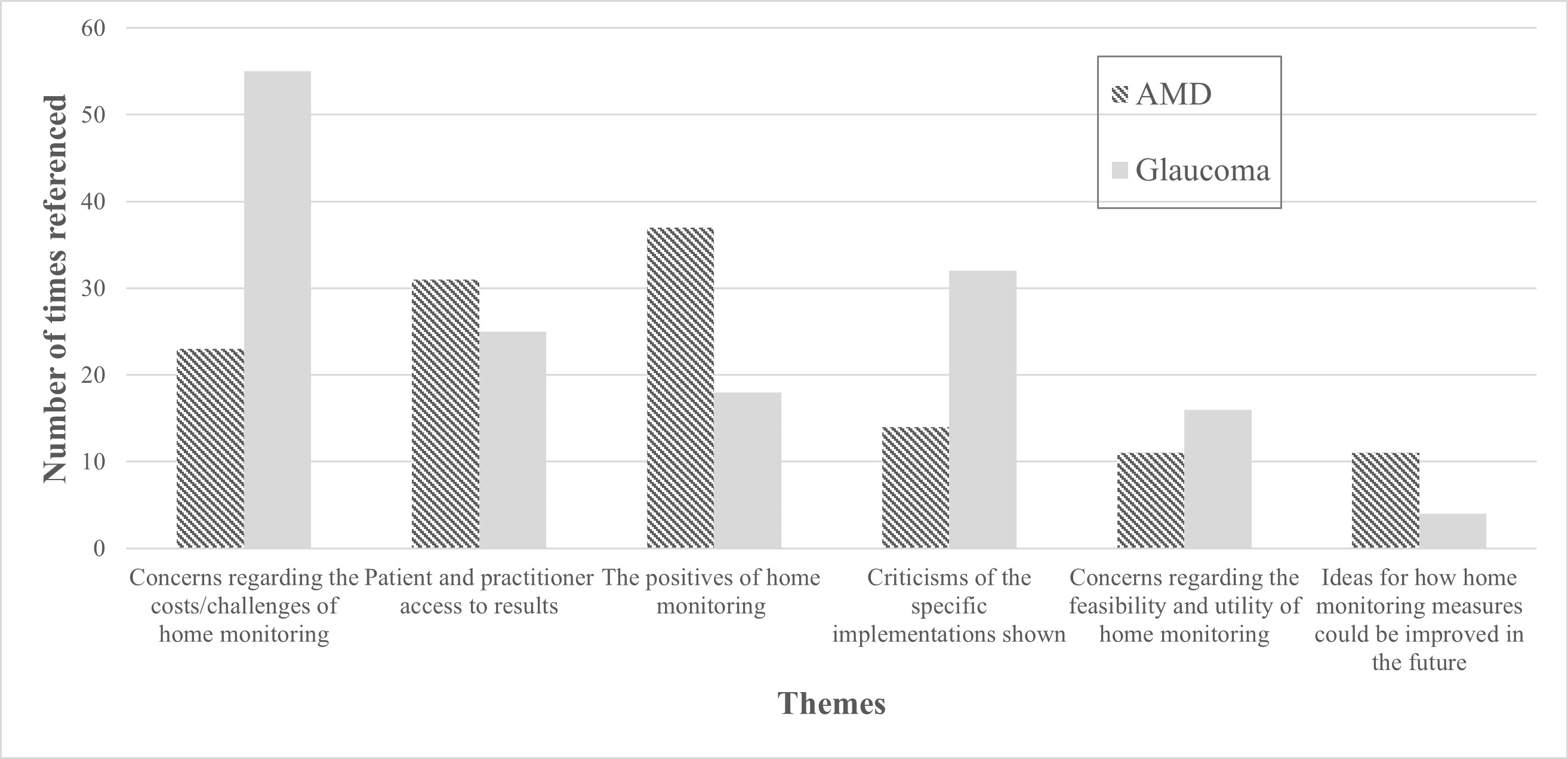

Supplement: online supplemental file 6 [file bmjopen-14-7-s006.png]
