# Supplementary material for: Views and opinions of patients with glaucoma and age-related macular degeneration on vision home-monitoring: a UK-based focus group study
Source: BMJ Open. 2024 Jul 12;14(7):e080619. doi: 10.1136/bmjopen-2023-080619 (PMC11253750; doi:10.1136/bmjopen-2023-080619)
Supplement: Supplementary file 7 [file bmjopen-2023-080619.reviewer_comments.pdf]

PEER REVIEW HISTORY

BMJ Open publishes all reviews undertaken for accepted manuscripts. Reviewers are asked to complete a checklist review form (**http://bmjopen.bmj.com/site/about/resources/checklist.pdf**) and are provided with free text boxes to elaborate on their assessment. These free text comments are reproduced below.

ARTICLE DETAILS

**TITLE (PROVISIONAL)**	The views and opinions of glaucoma and age-related macular degeneration patients on vision home-monitoring: A UK-based focus group study
**AUTHORS**	Dave, Sonali; Rathore, Mehal; Campbell, Peter; Edgar, David; Crabb, David; Callaghan, Tamsin; Jones, Pete

VERSION 1 – REVIEW

**REVIEWER**	Hogg, Henry<br>Newcastle University Faculty of Medical Sciences, Population Health Science Institute
**REVIEW RETURNED**	28-Oct-2023

**GENERAL COMMENTS**	Thank you for the opportunity to review this article. I think it represents important and scarce work to make patient ophthalmology perspectives accessible in the literature and is very timely for home vision monitoring as an intervention, which is now at a stage where its niche within healthcare pathways urgently requires clearer definition.<br><br>The flip side of this article's great strength as one of the few pieces of qualitative work in the literature is that it's target audience are unfamiliar with the approach and so I think a few alterations may benefit the authors in maximising their impact.<br><br>- implicit or explicit attempts to attribute significance to the frequency with which participants mentioned certain themes or sentiments in focus groups is misleading. As such I would suggest standardising the size of circles in Figures 2 and 3, removing figure 6 and adjusting the text accordingly. Such analysis should be framed within a subsequent survey if the authors see it as important, the role of interviews/focus groups here is to explore phenomena, not quantify or prioritise them.<br><br>- Although the SRQR checklist suggests that authors have shared their research paradigm and approach on pages 5 and/or 6 I cannot see where. I think this is very important due to the positivist paradigm under which the vast majority of this paper's audience operate.<br><br>- To assist readers in interpreting findings it is important to understand the roles and backgrounds of each of the research team. Who had the greatest impact on analysis/interpretation? What are the backgrounds of team members; clinicians, academics, technologists, hybrid roles etc.?

	- There are a few occasions where language around interviewing and focus groups appears blurred. As I understand it, no interviews were conducted so I suggest removing the word altogether to avoid confusion. My understanding is that focus groups were facilitated using a topic guide and there was a separate survey.<br><br>Thanks again or the opportunity to review and congratulations to the authors on an important and very well-written piece of work.

**REVIEWER**	OKONKWO, OGUGUA<br>Eye Foundation Hospital, Lagos, Ophthalmology
**REVIEW RETURNED**	28-Oct-2023

**GENERAL COMMENTS**	In general, the authors have used focused group discussion to highlight or determine the patient's perception of home monitoring using telemedicine. There is a sense of subjectivity to the methodology, in the sense that results may not be reproducible using an entirely different set of patients from somewhere else. However, important take homes that can influence the utility of home monitoring can be derived from the manuscript.<br>Abstract<br>Page 4: Line 45: Replace “people” with “patients living with glaucoma….”<br>I am not sure why the exclusion criteria include “if they lived further than a one-hour travel distance from the university.” Please give a clear reason for this exclusion criteria in the manuscript.<br><br><br>Introduction<br>Methods: Participants: “a self-reported a diagnosis of glaucoma or AMD, an….” Please delete “a” after “reported.”<br>A study limitation is that the patients were guided and not in real-life telemedicine situations or use. Also, the patient's pre-conceived ideas for or against telemedicine or technology could affect the feedback.<br>The study sample could be increased to achieve more representation. Was a sample size estimation done to determine the size required to make the findings more representative?<br><br>Analysis:<br>Line 459: “Five participants replied, all of whom responded positively, indicating that their view were well represented.”<br>Can the authors explain the low response rate, given that only 5 of 15 responses were received in this crucial step?<br><br>Results:<br>It is at the discretion of the authors to report which patient’s statement best aligns with the theme.<br>Statements from few patients appear to feature more. For instance, a majority of the statements cited for AMD discussions appeared to be from AMD Pt 1, and AMD Pt 2. The others contributed fewer statements to those statements reported. My guess is that bias was eliminated and dealt with by the objective analysis done. The spider diagram is quite illustrative and, in some ways, appears to show how the themes were derived. This is very helpful in understanding the output from the discussions.<br><br>Table S2: Age and year since diagnosis is not provided for patient Glaucoma 3. Please provide the reason for this observation.

	Discussion:<br>Line 485: change to “actually”<br><br>Line 512: I am not sure what n=17 and n=37 mean.<br><br>The idea of home monitoring is early detection of vision change. A change in vision in AMD is likely to be more acutely felt since it impacts central vision compared to glaucoma, which impacts mid-peripheral vision (on visual fields) and only affects central vision in the progression to advanced disease. Therefore, the awareness of vision change experienced by the patient is quite different for the two diseases.<br>Though already mentioned by the researchers, an important source of bias could be in the selection of the participants, who are urban and motivated patients. This may influence the direction of the discussion differently, were the sampled patients to be less motivated. It would appear reasonable in future studies to categorize the patient groups using factors that could influence the reporting by the patients, including if the patient is tech savvy or not, financial status, literacy level, and support available to the patients. Other demographics, including age and where he or she lives, will also be most helpful.<br><br>References<br>Appropriate references have been cited in the manuscript and adequately done.

VERSION 1 – AUTHOR RESPONSE

Reviewer: 1
Dr. Henry Hogg, Newcastle University Faculty of Medical Sciences, Newcastle Upon Tyne Hospitals NHS Foundation Trust
Comments to the Author:
Thank you for the opportunity to review this article. I think it represents important and scarce work to make patient ophthalmology perspectives accessible in the literature and is very timely for home vision monitoring as an intervention, which is now at a stage where its niche within healthcare pathways urgently requires clearer definition.

The flip side of this article's great strength as one of the few pieces of qualitative work in the literature is that it's target audience are unfamiliar with the approach and so I think a few alterations may benefit the authors in maximising their impact.

- implicit or explicit attempts to attribute significance to the frequency with which participants mentioned certain themes or sentiments in focus groups is misleading. As such I would suggest standardising the size of circles in Figures 2 and 3, removing figure 6 and adjusting the text accordingly. Such analysis should be framed within a subsequent survey if the authors see it as important, the role of interviews/focus groups here is to explore phenomena, not quantify or prioritise them.

We would like to thank the reviewer for these helpful comments. We assume from the text that the reviewer is referring to Figure 5 as there is no Figure 6 in the paper. Figure 5 and references to it

have been removed from the text. Additionally, the circle sizes in figures 2 and 3 have been standardized.

- Although the SRQR checklist suggests that authors have shared their research paradigm and approach on pages 5 and/or 6 I cannot see where. I think this is very important due to the positivist paradigm under which the vast majority of this paper's audience operate.

Thank you for your comment. This has been clarified on page 6, lines 88-90.

Page 6, lines 88-90*: The present focus group study used a positivist, qualitative study approach, featuring semi-structured topic guides, and facilitated by authors SD and MR.*

- To assist readers in interpreting findings it is important to understand the roles and backgrounds of each of the research team. Who had the greatest impact on analysis/interpretation? What are the backgrounds of team members; clinicians, academics, technologists, hybrid roles etc.?

We have given details of Authors' Contributions at the end of the paper. However, in response to this comment we have provided further details of the authors' backgrounds and their input to the paper in Supplemental material.

- There are a few occasions where language around interviewing and focus groups appears blurred. As I understand it, no interviews were conducted so I suggest removing the word altogether to avoid confusion. My understanding is that focus groups were facilitated using a topic guide and there was a separate survey.

We thank the reviewer for this helpful comment. We have now removed the term 'interview' from the manuscript and clarified that the study was a facilitated focus group design.

Page 6, lines 88-90: *This focus group study applied a positivist, qualitative study approach which used semi-structured topic guides, facilitated by authors SD and MR.*

Page 6, line 91: *Focus groups also encourage participants to engage in open conversation*

Page 6, lines 93-94: *A copy of the topic guide for the focus groups can be found as supplementary material (table S1)*

Thanks again or the opportunity to review and congratulations to the authors on an important and very well-written piece of work.

Thank you.

Reviewer: 2
Dr. OGUGUA OKONKWO, Eye Foundation Hospital, Lagos
Comments to the Author:
In general, the authors have used focused group discussion to highlight or determine the patient's perception of home monitoring using telemedicine. There is a sense of subjectivity to the methodology, in the sense that results may not be reproducible using an entirely different set of patients from somewhere else. However, important take homes that can influence the utility of home monitoring can be derived from the manuscript.

Thank you for your helpful comments which we have addressed below.

Abstract

Page 4: Line 45: Replace “people” with “patients living with glaucoma….”

Thank you. This change has now been made.

Page 3, line 2-3: *To investigate the views, hopes, and concerns of patients living with glaucoma and AMD regarding vision home-monitoring.*

I am not sure why the exclusion criteria include “if they lived further than a one-hour travel distance from the university.” Please give a clear reason for this exclusion criteria in the manuscript.

Thank you for your comment. This has now been clarified in the abstract.

Page 3, lines 13-14: *one hour travel distance from the University (to ensure minimal travel burden on participants).*

Introduction

Methods: Participants: “a self-reported a diagnosis of glaucoma or AMD, an….” Please delete “a” after “reported.”

Thank you. This change has now been made.

Page 6, line 74: *Inclusion criteria were: (1) a self-reported diagnosis of glaucoma or AMD*

A study limitation is that the patients were guided and not in real-life telemedicine situations or use. Also, the patient's pre-conceived ideas for or against telemedicine or technology could affect the feedback.

We agree that the study did not replicate a real-life telemedicine situation and that this is a limitation, and we address this issue in the “Strengths and Limitations of the Study” section on P4 where we state as a limitation that:- “ The study also took place in a university setting under supervision from the researchers, which doesn’t represent the real-life aspect of home monitoring, where patients would be expected navigate the tests relatively independently.” We also refer to this limitation in the final para on P22 in the “Limitations and Future work” section of the Discussion.

We accept that the patients’ pre-conceived ideas for or against telemedicine or technology could affect the feedback, and we acknowledge this as a limitation as regards technology on P4:- “However, the generalisability of the study is still limited by the fact that the participants were all volunteers with access to technology (as they were contacted by email)” and this limitation is repeated on page 21 in the “Limitations and Future Work” section of our Discussion. While it is undoubtedly correct that the participants’ pre-conceived ideas on telemedicine could affect their feedback, we believe this is a factor inherent to feedback from any study seeking participants’ views on telemedicine. The bigger and more random the sample the more likely it is that the participants will reflect the full range of pre-conceived ideas on telemedicine, but this is not achievable with our small sample which limits the generalisability of our findings. We highlight this lack of generalisability in the first sentence of our “Limitations and Future Work” on P21 which states:- “The primary limitations of this study relate to generalisability of the sample population.”

The study sample could be increased to achieve more representation. Was a sample size estimation done to determine the size required to make the findings more representative?
Thank you for your comment. There was no formal sample size calculation. After the second focus

group, no new themes emerged and the research team decided that data saturation had been reached. Previous literature suggests that reaching data saturation captures the breadth of issues, even though there were only a few focus groups (1). The literature also suggests that 80% of saturation can be found within 2 or 3 groups (2). It can be argued that the data collected within the 4 focus groups were detailed enough to capture the patient opinions and experiences. Therefore, the team decided that this sample size was adequate. However, we do acknowledge that this group of participants does not represent the general population with glaucoma, and we state this on P4 and P21 as noted above, but the information does provide helpful insights into the patient experience.

1. Hennink MM, Kaiser BN, Weber MB. What Influences Saturation? Estimating Sample Sizes in Focus Group Research. Qual Health Res. 2019;29(10):1483-96.
2. Guest G, Namey E, McKenna K. How Many Focus Groups Are Enough? Building an Evidence Base for Nonprobability Sample Sizes. Field Methods. 2017;29(1):3-22.

Analysis:
Line 459: "Five participants replied, all of whom responded positively, indicating that their view were well represented."
Can the authors explain the low response rate, given that only 5 of 15 responses were received in this crucial step?
Unfortunately, although all participants were contacted and followed up, only 5 participants reviewed the manuscript. However, in the outreach email, participants were told that they did not have to respond if they did not have any comments. Therefore, it was assumed that none of the remaining participants had any comments.

Results:
It is at the discretion of the authors to report which patient's statement best aligns with the theme. Statements from few patients appear to feature more. For instance, a majority of the statements cited for AMD discussions appeared to be from AMD Pt 1, and AMD Pt 2. The others contributed fewer statements to those statements reported. My guess is that bias was eliminated and dealt with by the objective analysis done. The spider diagram is quite illustrative and, in some ways, appears to show how the themes were derived. This is very helpful in understanding the output from the discussions.
Thank you for your comment. The analysis of the statements was completed by two other authors (SD and PJ) and all authors reviewed the manuscript to ensure there was no bias in the selection of statements used. In these focus groups, there were some participants who contributed more than others which would explain the variability in the number of statements presented per participant.
Table S2: Age and year since diagnosis is not provided for patient Glaucoma 3. Please provide the reason for this observation.
This participant was contacted for their information but they did not respond. They were contacted a total of three times so unfortunately, we were not able to include this information. This information has been added to the table caption for supplemental table S3.

Page 30, lines 727-729: *Table S3: Table showing participant demographic and diagnosis information. Glaucoma participant 3 was unable to be contacted for their age and therefore this information could not be presented.*

Discussion:
Line 485: change to "actually"
Thank you. This change has now been made.
Line 512: I am not sure what n=17 and n=37 mean.

Thank you for your comment. These numbers represent the how many references were made for these two themes. However, these numbers have now been removed from the text to avoid confusion.

Page 21, lines 478-479: *Participants with glaucoma discussed the positives of home-monitoring less often than those in the AMD groups.*

The idea of home monitoring is early detection of vision change. A change in vision in AMD is likely to be more acutely felt since it impacts central vision compared to glaucoma, which impacts mid-peripheral vision (on visual fields) and only affects central vision in the progression to advanced disease. Therefore, the awareness of vision change experienced by the patient is quite different for the two diseases.

This is an important point and indeed was a driver in our decision to include both AMD and Glaucoma patients in our sample. As we note in our Introduction (bottom of page 5):- “And we included both glaucoma and AMD patients, to try to generalise beyond a single patient population.”

Though already mentioned by the researchers, an important source of bias could be in the selection of the participants, who are urban and motivated patients. This may influence the direction of the discussion differently, were the sampled patients to be less motivated. It would appear reasonable in future studies to categorize the patient groups using factors that could influence the reporting by the patients, including if the patient is tech savvy or not, financial status, literacy level, and support available to the patients. Other demographics, including age and where he or she lives, will also be most helpful.

We couldn’t agree more! However, the editorial guidance on this topic of demographics states:- “As a general rule, we allow a maximum of two indirect identifiers in a table (e.g., age range and sex).” This allowance, though fully understandable, does limit authors somewhat in terms of the demographic information that can be included.
